# Supplementary material for: The European Thyroid Imaging and Reporting Data System as a Remedy for the Overdiagnosis and Overtreatment of Thyroid Cancer: Results from the EUROCRINE Surgical Registry
Source: Cancers (Basel). 2024 Jun 17;16(12):2237. doi: 10.3390/cancers16122237 (PMC11202303; doi:10.3390/cancers16122237)
Supplement: Supplementary file 1 [file cancers-16-02237-s001.zip › cancers-3038383-supplementary.pdf]

Supplementary Materials - Index

Table S1 Diagnostic performance of EU-TIRADS by individual cutoffs.

**Cutoff for malignancy**	**AUC**	**ACC**	**SEN**	**SPE**	**PLR**	**NLR**	**PPV**	**NPV**
EU-TIRADS ≥ 2	0.538	0.431	0.988	0.088	1.084	0.133	0.400	0.924
EU-TIRADS ≥ 3	0.607	0.524	0.958	0.257	1.289	0.165	0.442	0.908
EU-TIRADS ≥ 4	0.687	0.646	0.857	0.517	1.773	0.277	0.522	0.854
EU-TIRADS ≥ 5	0.722	0.758	0.571	0.872	4.457	0.492	0.733	0.768

AUC = Area Under Curve, ACC = accuracy, SEN = sensitivity, SPE = specificity, PLR = positive likelihood ratio, NLR = negative likelihood ratio, PPV = positive predictive value, NPV = negative predictive value.
